# Evidence for Contemporary Switching of the O-Antigen Gene Cluster between Shiga Toxin-Producing *Escherichia coli* Strains Colonizing Cattle

**DOI:** 10.3389/fmicb.2017.00424

**Published:** 2017-03-21

**Authors:** Lutz Geue, Christian Menge, Inga Eichhorn, Torsten Semmler, Lothar H. Wieler, Derek Pickard, Christian Berens, Stefanie A. Barth

**Affiliations:** ^1^Friedrich-Loeffler-Institut/Federal Research Institute for Animal Health, Institute of Molecular PathogenesisJena, Germany; ^2^Institute of Microbiology and Epizootics, Free University BerlinBerlin, Germany; ^3^Robert Koch InstituteBerlin, Germany; ^4^Wellcome Trust Sanger Institute, Pathogen GenomicsCambridge, UK

**Keywords:** *E. coli*, STEC, O-antigen gene cluster, recombination, genome sequencing

## Abstract

Shiga toxin-producing *Escherichia coli* (STEC) comprise a group of zoonotic enteric pathogens with ruminants, especially cattle, as the main reservoir. O-antigens are instrumental for host colonization and bacterial niche adaptation. They are highly immunogenic and, therefore, targeted by the adaptive immune system. The O-antigen is one of the most diverse bacterial cell constituents and variation not only exists between different bacterial species, but also between individual isolates/strains within a single species. We recently identified STEC persistently infecting cattle and belonging to the different serotypes O156:H25 (*n* = 21) and O182:H25 (*n* = 15) that were of the MLST sequence types ST300 or ST688. These STs differ by a single nucleotide in *purA* only. Fitness-, virulence-associated genome regions, and CRISPR/CAS (clustered regularly interspaced short palindromic repeats/CRISPR associated sequence) arrays of these STEC O156:H25 and O182:H25 isolates were highly similar, and identical genomic integration sites for the *stx* converting bacteriophages and the core LEE, identical Shiga toxin converting bacteriophage genes for *stx1a*, identical complete LEE loci, and identical sets of chemotaxis and flagellar genes were identified. In contrast to this genomic similarity, the nucleotide sequences of the O-antigen gene cluster (O-AGC) regions between *galF* and *gnd* and very few flanking genes differed fundamentally and were specific for the respective serotype. Sporadic aEPEC O156:H8 isolates (*n* = 5) were isolated in temporal and spatial proximity. While the O-AGC and the corresponding 5′ and 3′ flanking regions of these aEPEC isolates were identical to the respective region in the STEC O156:H25 isolates, the core genome, the virulence associated genome regions and the CRISPR/CAS elements differed profoundly. Our cumulative epidemiological and molecular data suggests a recent switch of the O-AGC between isolates with O156:H8 strains having served as DNA donors. Such O-antigen switches can affect the evaluation of a strain's pathogenic and virulence potential, suggesting that NGS methods might lead to a more reliable risk assessment.

## Introduction

Shiga toxin-producing *Escherichia coli* (STEC) comprise a group of zoonotic enteric pathogens (Nataro and Kaper, 1998). The main reservoirs for STEC strains are ruminants, with cattle in particular. In humans, STEC infection may result in diarrhea, frequently complicated by the onset of hemorrhagic colitis (HC), or several renal and neurological sequelae, including the hemolytic uremic syndrome (HUS; Griffin and Tauxe, 1991; Su and Brandt, 1995; Paton and Paton, 1998; Remuzzi and Ruggenenti, 1998). Factors contributing to the virulence of STEC strains causing human disease, also referred to as enterohemorrhagic *E. coli* (EHEC), include two major phage-encoded toxins, Shiga toxin 1 (Stx1) and 2 (Stx2), which can be produced and secreted by different strains individually or in combination. STEC may additionally possess virulence characteristics such as the ability to cause attaching-and-effacing (AE) lesions in the large intestine (McKee et al., 1995), and a large plasmid encoding for an enterohemolysin (*hlyA/ehxA*), a catalase-peroxidase (*katP*), and an extracellular serine protease (*espP*; Schmidt et al., 1995; Brunder et al., 1996, 1997, 1999).

Lipopolysaccharide (LPS), a major component of the outer membrane, represents the principal virulence factor of gram-negative bacteria (reviewed in Lerouge and Vanderleyden, 2002). It consists of three distinct regions: lipid A, core oligosaccharide, and O-specific polysaccharides (O-antigens; Lerouge and Vanderleyden, 2002; Samuel and Reeves, 2003). While lipid A is the main driver of inflammatory responses, O-antigens are instrumental for host colonization, and bacterial niche adaptation (Reeves, 1995). O-antigens are highly immunogenic and, therefore, targeted by the adaptive immune system. Recognition by antibodies, e.g., initiates the classical complement pathway resulting in bacterial cell death or increased phagocytosis by cells of the host defense (Reeves, 1995). Due to this strong selective pressure, the O-antigen is one of the most variable bacterial cell constituents, with variation in the types of sugars present, their arrangement within the O-unit, and the linkages between O-units (Wang et al., 2001; Bazaka et al., 2011). Variation not only exists between different bacterial species, but also between individual clones within a single species (Penner and Aspinall, 1997; Stenutz et al., 2006; Lam et al., 2011; Liu et al., 2014). The existence of more than 180 O-antigens has been proposed so far for *E. coli* (Wang et al., 2001). This high variability is made use of in clinical and food microbiology and in epidemiology by applying serotyping for infection chain tracing and risk assessment of STEC strains isolated from patients or food for human consumption.

Three mechanisms for biosynthesis of the O-antigen seem to exist (Samuel and Reeves, 2003). The one most frequently employed by *E. coli* is the Wzy/Wzx-dependent pathway. The genes involved in O-antigen biosynthesis generally form an O-antigen gene cluster (O-AGC) in the chromosome. In *E. coli*, this cluster is flanked by the colanic acid biosynthesis gene cluster (*wca* genes) and the histidine biosynthesis (*his*) operon (Iguchi et al., 2015). The sequences of many O-AGC were used to determine the genetic basis of O-antigen evolution (Samuel and Reeves, 2003; Samuel et al., 2004). The results show that the O-AGC sections located between the *gnd* and *galF* genes have G+C contents lower (usually <40% in *E. coli* and *Salmonella enterica*) compared to the usual 51% genome average G+C content (Samuel et al., 2004). This atypical G+C content indicates that the O-AGC was acquired by interspecies horizontal gene transfer (HGT). Such an interspecies exchange was described for the O8 and O9 O-antigens of *E. coli*, as they are identical to O5 and O3 of *Klebsiella pneumoniae*, respectively (Sugiyama et al., 1997). An intraspecies switch in *Vibrio cholerae* from serogroup O1 to the novel serotype O139 was proposed to have been the initial event at the advent of a cholera epidemic in Asia (Mekalanos et al., 1997). This strain arose by HGT from a strain closely related to the pandemic *V. cholerae* O1 El Tor (Bik et al., 1995). Likewise, the acquisition in *E. coli* of the O157 O-AGC by an O55:H7 strain to generate the O157:H7 clone is considered critical for the evolution of this pandemic food-borne pathogen (Tarr et al., 2000; Wang et al., 2002). Besides having potential implications for the host range and virulence of clones of gram-negative bacteria, serotype switches interfere with serotype-based epidemiologic approaches to unveil infection chains and may even impact on the reliability of diagnostic workflows when these are shaped by serotype-based risk assessments. Here, we present evidence by integrating epidemiological and whole genome sequence data that an O-AGC cluster switch recently occurred between STEC and atypical enteropathogenic *E. coli* (aEPEC) clones of the serotypes O182:H25, O156:H25, and O156:H8 in a cattle herd (Geue et al., 2002; Barth et al., 2016).

## Materials and methods

### Bacterial isolates

During a longitudinal study investigating the prevalence of Shiga toxin-producing *E. coli* (STEC) in cattle (Geue et al., 2002), STEC isolates belonging to the serotypes O156:H25 (*n* = 21) and O182:H25 (*n* = 15) were isolated (Table 1). Isolates of the serotype O156:H25 were present in three of four different herds tested (groups from farms B, C, and D1/D2; Geue et al., 2010), similar to isolates of serotype O182:H25 (groups from farms A, B, and D1/D2). Time periods in which STEC of both serotypes were isolated overlapped in farms B and D1/D2. Additionally, STEC isolates of serotype O156:H8 (*n* = 5) were found in farms A, B and D2 (Table 1). Other STEC isolates with H25 chemotaxis and flagella genes (O51:H25 [*n* = 1], O153:H25 [*n* = 3], O165:H25 [*n* = 22], O172:H25 [*n* = 4], O177:H25 [*n* = 6], ONT:H25 [*n* = 1]), that had been isolated during the same longitudinal study, were included in the current study.

**Table 1:** **Overview of strains included in this study, their origin, time of isolation and selected genetic properties**.

												**Same locus**						**Same locus**	
**Isolate**	**Serotype**	**Sequence type**	**Farm**	**No. of animal [DD.MM.YYYY]**	**Sampling day**	***stx* phage**	**Phage integration site**	***eae* subtype**	**LEE integration site**	**T2SS**	***espZ*-9 *nuc*-insertion**	***efa1*/*lifA*-like**	**AidA-I adhesin like**	***bcsA* gene insertion of 84 nt**	**10kb island phage like**	**38.4 kb phage (for ImpK, OmpA)**	***cad* operon (10 kb)**	***pgaABCD* operon (biofilm adhesin)**	***efeUOB* operon (low pH iron transporter)**
13E0602	O182:H25	300	A	9	23.02.1998	1a	*mlrA*/*yheU*	ζ	*pheU/pheV*	+	−	−	−	−	−	−	−	+	+
13E0644	O182:H25	300	A	26	23.03.1998	1a	*mlrA*/*yheU*	ζ	*pheU/pheV*	+	−	−	−	−	−	−	−	+	+
13E0646	O182:H25	300	A	26	23.03.1998	1a	*mlrA*/*yheU*	ζ	*pheU/pheV*	+	−	−	−	−	−	−	−	+	+
13E0647	O182:H25	300	A	26	23.03.1998	1a	*mlrA*/*yheU*	ζ	*pheU/pheV*	+	−	−	−	−	−	−	−	+	+
13E0648	O182:H25	300	A	26	23.03.1998	1a	*mlrA*/*yheU*	ζ	*pheU/pheV*	+	−	−	−	−	−	−	−	+	+
13E0701	O182:H25	300	B	4	16.03.1998	1a	*mlrA*/*yheU*	ζ	*pheU/pheV*	+	−	−	−	−	−	−	−	+	+
13E0725	O182:H25	300	B	20	07.12.1998	1a	*mlrA*/*yheU*	ζ	*pheU/pheV*	−	+	+	+	−	−	−	−	+	+
13E0726	O182:H25	300	B	20	07.12.1998	1a	*mlrA*/*yheU*	ζ	*pheU/pheV*	−	+	+	+	−	−	−	−	+	+
13E0728	O182:H25	300	B	20	07.12.1998	1a	*mlrA*/*yheU*	ζ	*pheU/pheV*	−	+	+	+	−	−	−	−	+	+
13E0729	O182:H25	300	B	23	17.08.1998	1a	*mlrA*/*yheU*	ζ	*pheU/pheV*	−	+	+	+	−	−	−	−	+	+
13E0798	O182:H25	300	D1	11	20.07.1998	1a	*mlrA*/*yheU*	ζ	*pheU/pheV*	+	−	−	−	−	+	−	−	+	+
13E0799	O182:H25	300	D1	11	20.07.1998	1a	*mlrA*/*yheU*	ζ	*pheU/pheV*	−	+	+	+	−	−	−	−	+	+
13E0820	O182:H25	300	D1	17	30.03.1998	1a	*mlrA*/*yheU*	ζ	*pheU/pheV*	+	−	−	−	−	+	−	−	+	+
13E0862	O182:H25	300	D2	6	18.01.1999	1a	*mlrA*/*yheU*	ζ	*pheU/pheV*	+	−	−	−	−	−	−	−	+	+
13E0879	O182:H25	300	D2	12	15.02.1999	1a	*mlrA*/*yheU*	ζ	*pheU/pheV*	+	−	−	−	−	−	−	−	+	+
13E0702	O156:H25	300	B	4	16.03.1998	1a	*mlrA*/*yheU*	ζ	*pheU/pheV*	+	−	−	−	+	+	+	+	−	−
13E0730	O156:H25	300	B	23	17.08.1998	1a	*mlrA*/*yheU*	ζ	*pheU/pheV*	+	−	−	−	+	+	−	+	−	−
13E0732	O156:H25	300	B	23	17.08.1998	1a	*mlrA*/*yheU*	ζ	*pheU/pheV*	+	−	−	−	+	+	−	+	−	−
13E0733	O156:H25	300	B	23	17.08.1998	1a	*mlrA*/*yheU*	ζ	*pheU/pheV*	+	−	−	−	−	+	−	+	−	−
13E0753	O156:H25	688	B	25	10.11.1997	1a	*mlrA*/*yheU*	ζ	*pheU/pheV*	+	−	−	−	−	+	−	+	+	+
13E0755	O156:H25	688	B	25	10.11.1997	1a	*mlrA*/*yheU*	ζ	*pheU/pheV*	−	−	−	−	−	+	+	+	+	+
13E0757	O156:H25	688	B	25	10.11.1997	1a	*mlrA*/*yheU*	ζ	*pheU/pheV*	+	−	−	−	−	+	+	+	+	+
13E0758	O156:H25	300	B	25	27.07.1998	1a	*mlrA*/*yheU*	ζ	*pheU/pheV*	+	−	−	−	+	+	−	+	−	−
13E0759	O156:H25	688	B	25	27.07.1998	1a	*mlrA*/*yheU*	ζ	*pheU/pheV*	+	−	−	−	−	+	+	+	+	+
13E0764	O156:H25	300	B	25	09.11.1998	1a	*mlrA*/*yheU*	ζ	*pheU/pheV*	+	−	−	−	+	+	−	+	−	−
13E0776	O156:H25	688	B	26	08.09.1997	1a	*mlrA*/*yheU*	ζ	*pheU/pheV*	+	−	−	−	−	+	−	+	+	+
13E0778	O156:H25	688	B	28	06.07.1998	1a	*mlrA*/*yheU*	ζ	*pheU/pheV*	+	−	−	−	+	+	+	+	−	−
13E0780	O156:H25	300	B	28	06.07.1998	1a	*mlrA*/*yheU*	ζ	*pheU/pheV*	+	−	−	−	+	+	+	+	−	−
13E0781	O156:H25	300	B	28	06.07.1998	1a	*mlrA*/*yheU*	ζ	*pheU/pheV*	+	−	−	−	+	+	+	+	−	−
13E0801	O156:H25	300	D1	12	10.08.1998	1a	*mlrA*/*yheU*	ζ	*pheU/pheV*	+	−	−	−	+	+	+	+	−	−
13E0802	O156:H25	300	D1	12	10.08.1998	1a	*mlrA*/*yheU*	ζ	*pheU/pheV*	+	−	−	−	+	+	+	+	−	−
13E0803	O156:H25	300	D1	12	10.08.1998	1a	*mlrA*/*yheU*	ζ	*pheU/pheV*	+	−	−	−	+	−	+	−	−	−
13E0845	O156:H25	300	C	25	09.02.1998	1a	*mlrA*/*yheU*	ζ	*pheU/pheV*	+	−	−	−	+	+	+	−	+	+
13E0848	O156:H25	300	D2	2	18.01.1999	1a	*mlrA*/*yheU*	ζ	*pheU/pheV*	+	−	−	−	+	+	−	+	−	−
13E0850	O156:H25	300	D2	2	18.01.1999	1a	*mlrA*/*yheU*	ζ	*pheU/pheV*	+	−	−	−	+	+	−	+	−	−
13E0902	O156:H25	300	D2	25	12.04.1999	1a	*mlrA*/*yheU*	ζ	*pheU/pheV*	−	−	−	−	+	+	−	+	−	−
13E0685	O156:H8	327	A	29	23.03.1998	−	−	ϑ	*ileX*	+	−	−	−	+	−	−	+	+	+
13E0767	O156:H8	327	B	26	07.12.1998	−	−	ϑ	*ileX*	+	−	−	+	+	−	−	+	+	+
13E0775	O156:H8	327	B	26	07.12.1998	−	−	ϑ	*ileX*	+	−	−	+	+	−	−	+	+	+
13E0883	O156:H8	327	D2	12	31.05.1999	−	−	ϑ	*ileX*	+	−	−	+	+	−	−	+	+	+
13E0890	O156:H8	327	D2	23	15.03.1999	−	−	ϑ	*ileX*	+	−	−	+	+	−	−	+	+	+

### Whole genome sequencing

Genomic DNA of the *E. coli* isolates was prepared using the ZR fungal/bacterial DNA kit (Zymo Research Europe GmbH, Freiburg, Germany) from overnight cultures in Luria Bertani broth following the instructions of the manufacturer. The DNA concentration was determined spectrophotometrically at 260 nm and analyzed for fragmentation by 1% TBE agarose gel electrophoresis. All isolates were whole genome sequenced using Illumina MiSeq 300 bp paired-end sequencing and a coverage >40× was obtained. The sequence read data was first subjected to quality control using the NGS toolkit (Patel and Jain, 2012). Reads with a minimum of 70% of bases having a phred score of >20 were defined as high quality reads. *De novo* assembly of resulting high quality filtered reads into contiguous sequences (contigs and scaffolds) was achieved using CLC Genomics Workbench 8.0 (CLC bio, Aarhus, Denmark).

One strain per serotype (O182:H25 [13E0725], O156:H25 [13E0780], O156:H8 [13E0767]) was additionally whole genome sequenced on a PacBio RSII system (Pacific Biosciences, USA) by a commercial service provider (GATC Biotech, Konstanz, Germany) utilizing PacBio single-molecule real-time (SMRT) technology. Subsequent *de novo* assembly utilizing the HGAP3 protocol yielded a single polished contig with 200-fold average reference coverage. In order to ensure closed circle conformation of the bacterial chromosome, mapping, sequence analyses, and annotation were carried out using the commercial software package Geneious (version 9.1.6, Biomatters Ltd., Auckland, New Zealand). The whole genome alignments were performed by MAUVE analysis (version 2.3.1; Darling et al., 2004) as plugin in the Geneious software package.

A Maximum Likelihood tree (GTR+G+I substitution model, 1000 bootstraps) was calculated from 53 previously described genes associated with chemotaxis and flagella of flagellar serotype H25 (Sperandio, 2001; Niba et al., 2007; Table S1 in Supplemental Material) of eight different O-antigen serotypes (O51, O153, O156, O165, O172, O177, O182, ONT) in PAUP^*^ (version 4.0b10). The same genes of the flagellar serotype H8 were used as outgroup.

### Ethics statement

An Ethics Statement is not necessary. The isolates were obtained by non-invasive rectal swabs during a longitudinal study already published (Geue et al., 2002). No animal experiments were carried out for this study.

## Results and discussion

A previous study on STEC colonization in cattle herds identified specific STEC clones, which could be isolated from herds over extended periods of time and were therefore considered as persistently colonizing this animal reservoir (Geue et al., 2002; Barth et al., 2016). These strains expressed the flagellar serotype H25 but differed in O-antigen serotypes (O156, O165, O182). To assess the underlying genetic basis, we performed whole genome sequence analyses of these *E. coli* isolates. By MLST (Wirth et al., 2006), all O182:H25 and 15 of the O156:H25 isolates were assigned to ST300. The remaining 6 O156:H25 isolates were allocated to ST688. These STs differ from each other by a single nucleotide in *purA* only (Barth et al., 2016). By contrast, the O165:H25 isolates were classified as ST119 which is widely separated from ST300/ST688 (Barth et al., 2016).

**Figure 1:**
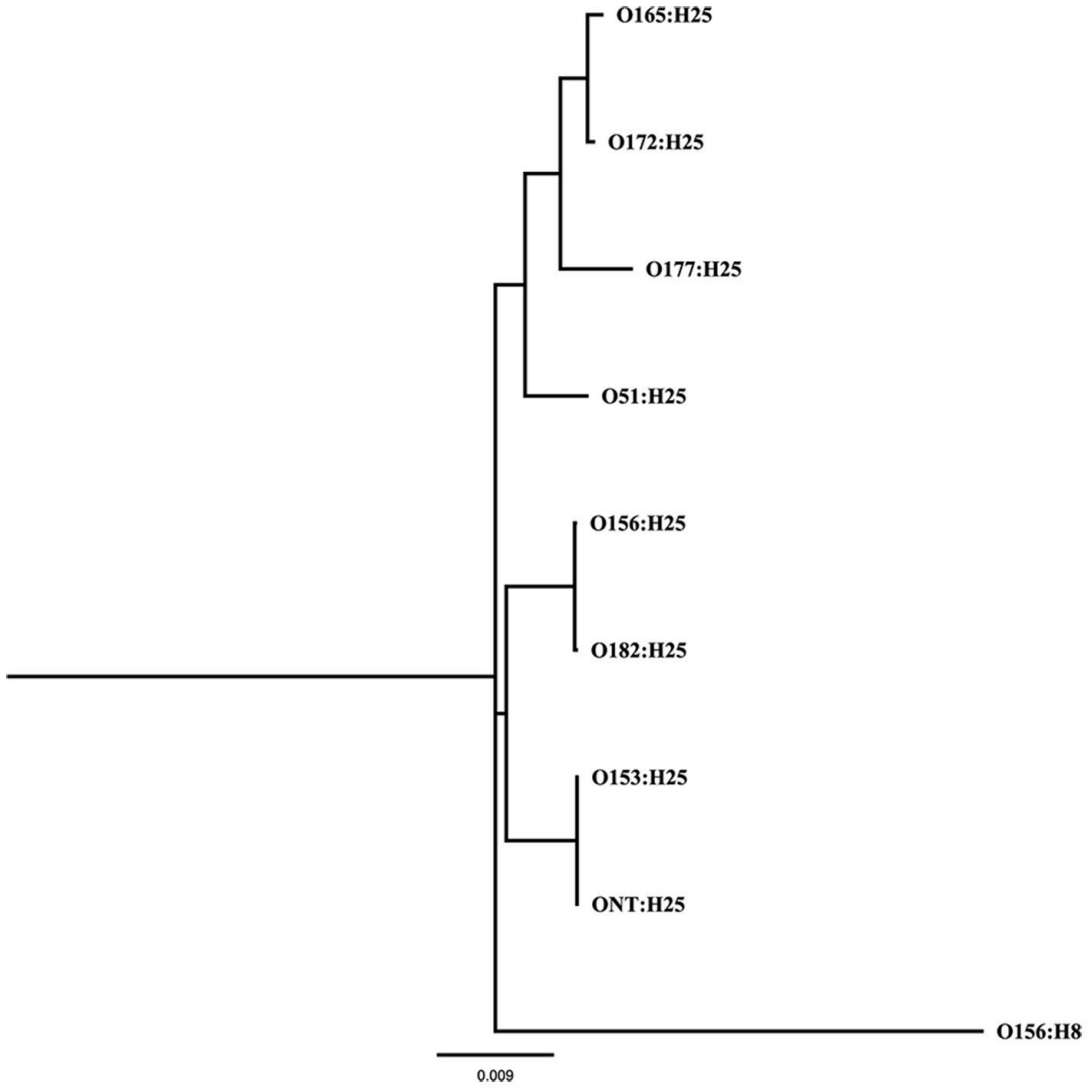
**Maximum Likelihood tree (GTR+G+I substitution model, 1,000 bootstraps) calculated from 53 genes associated with chemotaxis and flagella of flagellar serotype H25 of bovine STEC strains belonging to eight different O-antigen serotypes (O51, O153, O156, O165, O172, O177, O182, ONT); the same genes of the flagellar serotype H8 were used as outgroup**.

To further analyze that the genomic similarity of O156:H25 and O182:H25 isolates, we assessed the presence and relatedness of selected fitness- and virulence-associated genes. First, we compared 53 previously described genes associated with chemotaxis and flagella of H25 (Sperandio, 2001; Niba et al., 2007; Table S1 in Supplemental Material). A 100 % identity was detected for the nucleotide and amino acid sequences. Of the 45,429 nucleotides studied, only seven nucleotides differed between the strains of the two serotypes. They were located in the *flgD, flgI, flhA, fliG, fliZ, motA*, and *tar* genes and resulted in one amino acid exchange each in FlhA, FliG, and MotA, respectively. In contrast, H25 chemotaxis and flagellar genes from isolates belonging to other O serogroups (O51:H25, O153:H25, O165:H25, O172:H25, O177:H25, ONT:H25) exhibited larger genetic distances (between 97.2 and 99% identity, Figure 1). Additionally, a region spanning ~60 kb, which includes the complete core regions of the locus of enterocyte effacement (LEE) and the LEE insertions sites, was compared in all O156:H25 and O182:H25 isolates. The core regions were nearly identical (99.9–100%) in all isolates probed. Less than 40 nucleotides differed in the ~33,000 nucleotides considered. The main difference detected was a nine nucleotide insertion in the *espZ* gene of 5 of the 15 O182:H25, but in none of the O156:H25 isolates. Identical ζ *eae* genes were found in all O156:H25 and O182:H25 isolates and the LEE was inserted at the same *pheU*/*pheV* tRNA site in all isolates. The sequences of the 5′ and 3′ regions flanking the integration sites were identical in all O156:H25 and O182:H25 isolates. The *stx*-converting bacteriophages found in all O156:H25 and O182:H25 isolates encode the Stx subtype 1a and possess identical gene sequences for the A and B subunits. Phage genomes were all integrated between the *mlrA* (*yheY*) and *yheU* genes. In comparison to *E. coli* MG1655 (accession no. U00096.2), IAI1 (accession no. NC_011741.1), and HUSEC2011 (accession no. HF572917.2), the first 160 nucleotides of the coding sequence of the *mlrA* gene, associated with the regulation of curli synthesis in *E. coli* and *Salmonella enterica* (Brown et al., 2001), were lacking in all O156:H25 and O182:H25 isolates. Regarding the clustered, regularly interspaced, short palindromic repeat (CRISPR) acquired immune system, which is being used for determining the evolutionary divergence of *E. coli* isolates, especially for closely related strains (Touchon et al., 2011; Yin et al., 2013), we identified a set of identical CRISPR associated sequence type E (CAS-E) genes adjacent to the CRISPR2.1 locus (nomenclature as described by Diez-Villasenor et al., 2010) between the *cysH* and *iap* genes in all 21 O156:H25 isolates. The STEC O182:H25 isolate 13E0725, which had been sequenced by PacBio RSII, also contained a 100% identical CAS-E region. These CAS genes were also found in the other 14 O182:H25 isolates, but the quality of the Illumina sequence data was not sufficient to allow an unambiguous sequence assignment for the entire region. The 10 repeats and the 9 spacers of the CRISPR2.1 loci were 100% identical in all O156:H25 and O182:H25 isolates. An additional CRIPR2.2-3 array, lacking CAS genes, was detected between *queE (ygcF)* and *ygcE* in all O156:H25 and O182:H25 isolates. Here, too, the identity of the 7 repeats and the 6 spacers was 100% in all isolates. Besides the CRISPR2 loci, we also detected a CRISPR4.1-2 array situated between the genes encoding *clpA* and *infA*. This array also lacked CAS genes, but the entire 684 bp sequence between the stop codons of *infA* and *clpA* contained two repeats flanking a single spacer element. Its sequences were identical in the 21 O156:H25 and the 15 O182:H25 isolates and also in the *E. coli* reference strain MG1655 (accession no. U00096.2).

**Figure 2:**
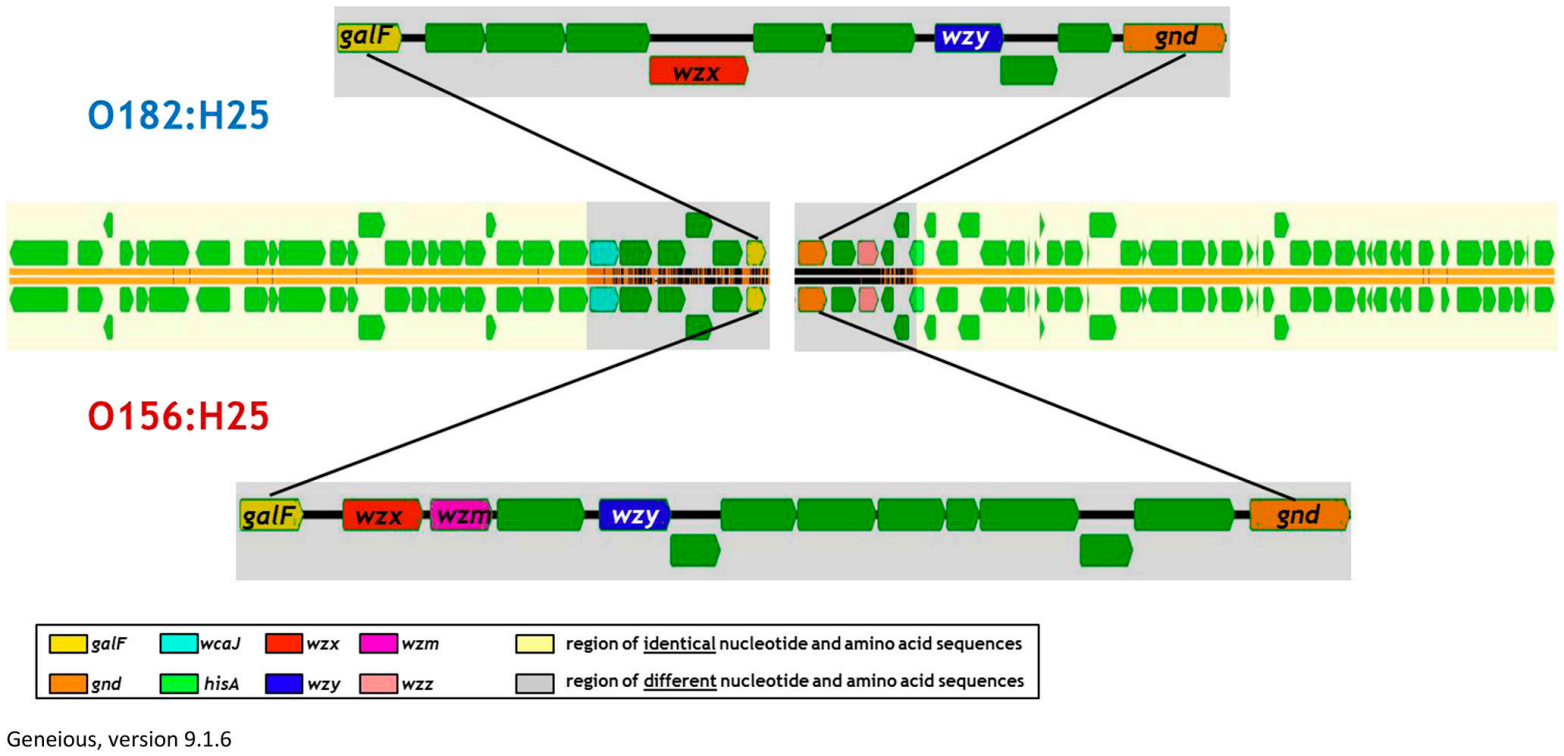
**Comparison of the architecture of the O-antigen gene clusters in bovine STEC strains of the O156:H25 (isolate 13E0780) and the O182:H25 (13E0725) serotype consecutively isolated on the same farm**.

When analyzing the O-AGC and the corresponding 5′ and 3′ flanking regions of the O156:H25 and O182:H25 isolates, it became apparent that both O-AGC serotypes deploy the Wzy/Wzx-dependent pathway. The G+C content of the O-AGC part located between the *galF* and *gnd* genes was <40% (35.8% for O156:H25 and 34.0% for O182:H25). Similar low G+C-values have been described previously by Samuel and coworkers for other *E. coli* and *Salmonella enterica* strains (Samuel et al., 2004). However, the nucleotide sequences of the regions between the *galF* and *gnd* genes were very different between the two serotypes (45.7% identity). The size of the O-AGC of the O156:H25 isolates amounted to 13,260 bp. In contrast, the region between *galF* and *gnd* of the O182:H25 isolates was only 9,861 bp long. Both the number and the order of genes differed substantially between the O-AGCs (Figure 2). Furthermore, the *galF* genes and the next five genes upstream of *galF* varied noticeably in their nucleotide sequences (between 9 and 59 different nucleotides) although the differences at the amino acid sequence level were considerably lower (only 1–5 amino acid exchanges, Figure 2, Table 2). Further upstream, all corresponding genes of the O156:H25 and O182:H25 isolates were identical. Likewise, the *gnd* genes and the next five genes downstream of *gnd* differed significantly in their nucleotide and amino acid sequences (between 7 and 182 nucleotides, 2–51 amino acids). Further downstream, all corresponding genes were again identical in the O156:H25 and O182:H25 isolates.

**Table 2:** **Comparison of nucleotide and amino acid sequences as well as the GC content of genes flanking the O-antigen cluster in bovine STEC strains belonging to the O156:H25 (***n*** = 21 strains) and the O182:H25 serotype (***n*** = 15 strains)**.

**No**.	**Gene**	**% Identity nucleotide sequence (no. of mismatches)**	**% GC content**	**% Identity amino acid sequence (no. of exchanges)**
1	*yegE*	100	53.8	100
2	*udk*	100	48.0	100
3	*dcd*	100	60.3	100
4	*asmA*	99.9 (1 in all isolates)	52.8–52.9	100
5	*yegH*	100	53.1	100
6	*wza*	99.9 (1 in all isolates)	53.8	100
7	*wzb*	99.8–100 (1 in 4 of 21 O156:H25 isolates)	54.2–54.3	99.3–100 (1 in 4 of 21 O156:H25 isolates)
8	*wzc*	100	52.5	100
9	*wcaA*	100	51.0	100
10	*wcaB*	99.8–100 (1 in 15 of 21 O156:H25 isolates)	53.1	100
11	*wcaC*	100	54.4	100
12	*wcaD*	100	42.4	100
13	*wcaE*	100	45.9	100
14	*wcaF*	100	47.5	100
15	*gmd*	100	55.1	100
16	*fcl*	100	56.3	100
17	*gmm*	100	55.7	100
18	*wcaI*	100	55.7	100
19	*cpsB*	99.9–100 (1 in 5 of 15 O182:H25 isolates)	55.3–55.4	99.8–100 (1 in 5 of 15 O182:H25 isolates)
20	*cpsG*	100	55.2	100
21	*wcaJ*	99.4 (9 in all isolates)	53.8	100
22	*wzxC*	97.3 (40 in all isolates)	54.1	99.2 (2 in all isolates)
23	*wcaK*	96.2 (49 in all isolates)	54.5	98.8 (4 in all isolates)
24	*wcaL*	95.2 (59 in all isolates)	54.5	99.0 (4 in all isolates)
25	*wcaM*	96.0 (56 in all isolates)	47.6	98.3 (5 in all isolates)
26	*galF*	96.5 (31 in all isolates)	50.7	99.7 (1 in all isolates)
**O-antigen gene cluster**				
27	*gnd*	95.4 (65 in all isolates)	50.6	99.6 (2 in all isolates)
28	UDP-glucose 6-dehydrogenase gene	86.7 (182 + gaps in all isolates)	43.6	89.2 (51 + gaps in all isolates)
29	*wzz*	94.1 (112 + gaps in all isolates)	47.7	97.5 (26 + gaps in all isolates)
30	*hisE*	96.6 (21 in all isolates)	53.3	98.5 (3 in all isolates)
31	*hisF*	97.2 (22 in all isolates)	52.2	98.8 (3 in all isolates)
32	*hisA*	99.1 (7 in all isolates)	56.6	99.6 (1 in all isolates)
33	*hisH*	100	56.3	100
34	*hisB*	100	53.7	100
35	*hisC*	100	54.7	100
36	*hisD*	100	57.1	100
37	*hisG*	100	54.6	100
38	*hisL*	100	43.1	100
39	*yefM*	100	47.6	100
40	*yoeB*	100	44.3	100
41	*yeeZ*	100	51.5	100
42	*yeeY*	100	50.6	100
43	*yeeF*	100	52.7	100
44	*yeeE*	100	50.5	100
45	Hypothetical protein gene (1)	100	51.3	100
46	*sbcB*	100	50.7	100
47	*dacD*	99.9–100 (1 in 7 of 15 O182:H25 isolates)	49.3	99.9–100 (1 in 7 of 15 O182:H25 isolates)
48	*sbmC*	100	46.0	100
49	*yeeA*	100	50.1	100
50	Hypothetical protein gene (2)	100	47.6	100
51	*yoeF*	100	45.0	100
52	*cobU*	100	50.8	100
53	*cobS*	100	53.5	100
54	*cobT*	100	52.6	100
55	*erfK*	100	49.9	100
56	Transcriptional regulator, DeoR family gene	100	44.7	100
57	Putative PTS IIA-like nitrogen-regulator protein PtsN gene	100	43.6	100
58	PTS system, gelactitol-specific IIB component gene	100	43.2	100
59	Sugar-phosphate isomerase, RpiB/LacA/LacB family gene	100	45.6	100
60	Transcriptional regulator, DeoR family gene 2	99.9–100 (1 in 14 of the 21 O156:H25 isolates), 99.7 (2 in 1 of O156:H25 isolates)	43.9–44.0	99.9–100 (1 in 14 of the 21 O156:H25 isolates), 99.7 (2 in 1 of O156:H25 isolates)
61	Putative PTS IIA-like nitrogen-regulatory protein PtsN gene 2	99.8 (1 in all isolates)	42.2	100
62	PTS system, galactitol-specific IIC component gene	100	43.5	100
63	Ribulose-phosphate3-epimerase gene	100	38	100
64	Short-chain dehydrogenase/reductase SDR gene	100	42.6	100
65	Sugar isomerase gene	100	43.3	100
66	*nac*	100	48.8	100

Taken together, the results of both core and accessory genome analyses based on Illumina sequence data proved a great degree of similarity between the genomes of the O156:H25 and the O182:H25 isolates. Only the genomic regions encoding the O-AGC between the *galF* and *gnd* genes and very few genes flanking these regions, but still part of the O-AGC, varied substantially and were specific for the respective serotype. This could suggest a possible switch of the O-AGC between isolates, and the epidemiological data substantiated this hypothesis, despite the O-AGC not having been specifically selected for in the original strain isolation (Geue et al., 2002). Isolates of both serotypes were isolated on the same farms and in identical sampling periods. On farm B, O156:H25 and O182:H25 isolates were even detected on the same day in the same group (March of the 2nd study year) and, on one occasion, also on the same day in the same animal (August of the 2nd study year; Table 1).

**Figure 3:**
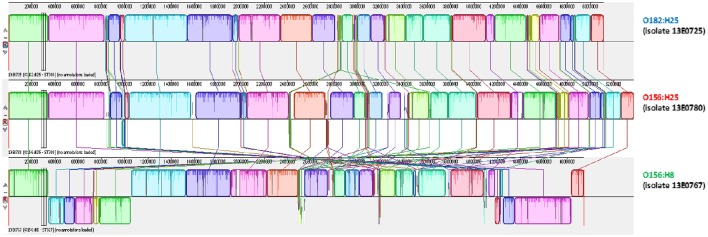
**MAUVE analysis of representative bovine STEC isolates of the O182:H25 (13E0725), the O156:H25 (13E0780), and the O156:H8 (13E0767) serotype consecutively isolated on the same farm**.

To further substantiate these findings, one isolate per serotype was randomly picked for 3rd generation whole-genome sequencing on a PacBio RSII system. Closed circle conformations of the bacterial chromosomes and their annotation were performed. By MAUVE analysis the arrangement of homologous sequence blocks was found to be very similar in both isolates (Figure 3; Darling et al., 2004). A difference was observed in the size of the genomes. The genome of O156:H25 isolate 13E0780 had a size of 5,371,291 nucleotides, whereas the one of O182:H25 isolate 13E0725 comprised of only 5,112,484 nucleotides. The difference was mainly due to the presence of an additional pathogenic island with a type II secretion system and an additional phage-like sequence in the STEC isolate 13E0780. A peculiarity of STEC isolate 13E0725 was the presence of a pathogenic island with an *efa1*/*lifA*-like gene and a gene encoding an AidA-I adhesion like protein. Upon inspection of the Illumina whole genome data of all STEC O182:H25 and STEC O156:H25 strains, the type II secretion system was missing in strain 13E0725 and in three other STEC O182:H25 strains, but it was also not present in 3 of the 21 STEC O156:H25 strains. In contrast, the pathogenicity island harboring an *efa1*/*lifA*-like gene and a gene for an AidA-I adhesion like protein was detected in only 5 of the 15 STEC O182:H25 strains and in none of the STEC O156:H25 strains (Table 1). From integrating molecular and epidemiological data it is tempting to conclude that a switch of the O-AGC might have occurred between the STEC O182:H25 and the O156:H25 clone present in the sampled cattle herd.

Noteworthy, five O156:H8 isolates were isolated during the same investigation period and on the same farms B and D. In contrast to the O156:H25 isolates, these isolates were typed as ST327 by MLST analysis. This sequence type is very different from ST300/688 (six of seven alleles different). Also, the virulence associated genome regions were significantly different from the O156:H25/O182:H25 isolates. The O156:H8 isolates are atypical enteropathogenic *E. coli* (aEPEC; Hernandes et al., 2009), lacking both *stx*-bacteriophages as well as *bfpA*. Also the LEE locus differed from the one present in the O156:25/O182:H25 isolates. For example, *eae* genes for a ϑ intimin were detected and the LEE of all O156:H8 isolates was inserted in the *ileX* tRNA site. As in the case of STEC O156:H25 and O182:H25, one random aEPEC O156:H8 isolate (13E0767) was whole-genome sequenced using 3rd generation sequencing. The sequences were aligned in a MAUVE analysis together with both STEC isolates. The orientation and the sequence of genome blocks varied distinctly in comparison to O156:H25/O182:H25 (Figure 3). With respect to the CRISPR/CAS systems in the five aEPEC O156:H8 isolates, most of the CAS gene array is missing in the CRISPR2.1 locus. These isolates have a deletion spanning from the second nucleotide of the codon encoding for Gly683 in the *cas3* gene to the last nucleotide (nt 29) of a CRISPR2 repeat element. The truncated CAS3 protein lacks 169 residues at its C-terminus and another C-terminal 48 residues are mutated due to the frameshift. The number of repeat and spacer elements also differed in contrast to the O156:H25/O182:H25 isolates. Only seven repeats and six spacers were found. Compared to the O156:H25/O182:H25 isolates, all O156:H8 isolates carried a deletion of ca. 12,000 nt ranging from *queD* right up to the CRISPR2.2-3 array. A smaller number of six repeats and five spacers was found in this locus. We also detected a CRISPR4.1-2 array in the O156:H8 isolates. Its two repeats are sequence-identical with the O156:H25/O182:H25 repeats, but the spacer's sequence differs.

**Figure 4:**
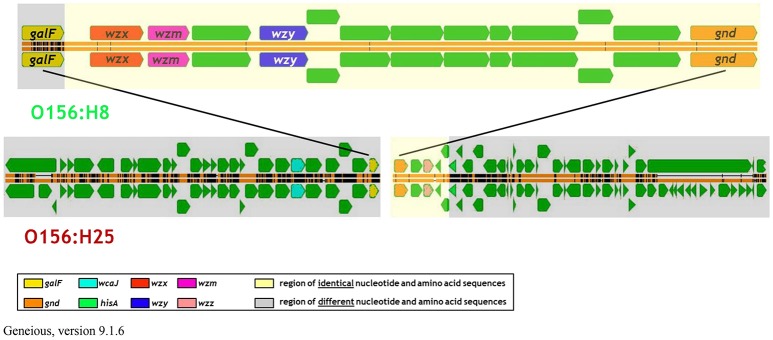
**Comparison of the architecture of the genome regions harboring the O-antigen gene cluster between bovine STEC strains belonging to the O156:H25 (isolate 13E0780) and the O156:H8 (13E0767) serotype consecutively isolated on the same farm**.

Analysis of the O-AGC and the corresponding 5′ and 3′ flanking regions of the O156:H8 isolates revealed that the region between *galF* and *gnd* of the O156:H8 isolates was identical to the respective region in the STEC O156:H25 isolates (Figure 4). Only 7 of the 14,154 nucleotides differed between the two serotypes in five different genes. Thereof, two nucleotide exchanges resulting in two amino acid exchanges were found in the *wzx* gene. One nucleotide exchange each was detected in the glycosyl transferase genes *wfeX, wfeY*, and in the *manB* gene. These nucleotide exchanges caused one amino acid exchange in WfeY. In contrast, the *galF* genes and all genes upstream of *galF* varied distinctly in their nucleotide and amino acid sequences (Figure 4, Table 3). The *gnd* genes and the next four genes downstream of *gnd* were identical in the O156:H8 and O156:H25 isolates. Further downstream in the genome, all genes differed substantially.

**Table 3:** **Comparison of nucleotide and amino acid sequences as well as the GC content of genes flanking the O-antigen cluster in bovine STEC strains belonging to the O156:H25 (***n*** = 21 strains) and the O156:H8 serotype (***n*** = 5 strains)**.

**No**.	**Gene**	**% Identity nucleotide sequence (no. of mismatches)**	**% GC content**	**% Identity amino acid sequence (no. of exchanges)**
1	*yegE*	Much smaller in H25 (probably two genes)	n.t.	n.t.
2	*udk*	99.2 (5 in all isolates)	48.4	99.5 (1 in all isolates)
3	*dcd*	99.5 (3 in all isolates)	60.2	100
4	*asmA*	98.7 (24 in all isolates)	52.6	99.8 (1 in all isolates)
5	*yegH*	98.8–99.0 (17 in 1 O156:H8 isolate, 16 in 4 other O156:H8 isolates)	58.2	99.8 (1 in all isolates)
6	*wza*	98.8 (14 in all isolates)	53.5	99.7 (1 in all isolates)
7	*wzb*	99.8 (1 in all O156:H8 isolates, 1 additional nucleotide in 4 of 21 O156:H25 isolates)	54.2	99.9–100 (1 in 4 of 21 O156:H25 isolates)
8	*wzc*	97.8 (47 in all isolates)	52.7	99.0 (7 in all isolates)
9	*wcaA*	98.0 (18 in 1 O156:H8 isolate, 17 in 4 other O156:H8 isolates)	51.1	99.3–99.6 (2 in 1 O156:H8 isolate, 1 in 4 other O156:H8 isolates)
10	*wcaB*	96.9 (14 in all O156:H8 isolates)	53.3	98.8 (2 in all isolates)
11	*wcaC*	99.3–99.5 (9 in 4 of 5 O156:H8 isolates, 6 in 1 other O156:H8 isolate)	54.1	99.5–99.8 (2 in 4 of 5 O156:H8 isolates, 1 in 1 other O156:H8 isolate)
12	*wcaD*	98.8 (15 in all isolates)	42.5	99.8 (1 in all isolates)
13	*wcaE*	97.1 (22 in all isolates)	45.9	98.4 (4 in all isolates)
14	*wcaF*	96.9–97.1 (16 in 1 O156:H8 isolate, 17 in 4 other O156:H8 isolates)	48.0	97.8–98.4 (3 in 1 O156:H8 isolate, 4 in 4 other O156:H8 isolates)
15	*gmd*	98.5–98.6 (16 in 1 O156:H8 isolate, 17 in 4 other O156:H8 isolates)	42.4	99.7 (1 in all isolates)
16	*fcl*	96.9 (30 in all isolates)	56.1	98.8 (4 in all isolates)
17	*gmm*	100	55.7	100
18	*wcaI*	99.4–99.5 (7 in 1 O156:H8 isolate, 6 in 4 other O156:H8 isolates)	55.7	99.5 (2 in all isolates)
19	*cpsB*	97.9 (30 in all isolates)	55.3	99.8 (1 in all isolates)
20	*cpsG*	96.9 (42 in all isolates)	55.2	98.9 (5 in all isolates)
21	*wcaJ*	97.2 (39 in all isolates)	53.7	99.4 (3 in all isolates)
22	*wzxC*	97.8 (32 in 4 O156:H8 isolates, 33 in 1 other isolate)	54.5	99.0 (5 in all isolates)
23	*wcaK*	97.7–97.8 (28 in 4 O156:H8 isolates, 29 in 1 other O156:H8 isolate)	54.6	98.8 (5 in 4 O156:H8 isolates, 6 in 1 other O156:H8 isolate)
24	*wcaL*	95.1 (60 in all isolates)	55.0	99.3 (3 in all isolates)
25	*wcaM*	98.5–98.6 (20 in 1 O156:H8 isolate, 21 in 4 other O156:H8 isolates)	47.4	98.7–98.9 (5 in 1 O156:H8 isolate, 6 in 4 other O156:H8 isolates)
26	*galF*	95.1 (44 in all isolates)	51.1	99.7 (1 in all isolates)
**O-antigen gene cluster**				
27	*gnd*	99.9–100 (1 in 4 O156:H8 isolates, 0 in 1 other O156:H8 isolate)	50.4	100
28	UDP-glucose 6-dehydrogenase gene	100	43.4	100
29	*wzz*	100	47.9	100
30	*hisE*	100	53.1	100
31	*hisF*	99.9 (1 in all isolates)	52.6	100
32	*hisA*	97.4 (19 in all isolates)	57.0	98.0 (5 in all isolates)
33	*hisH*	97.8 (13 in all isolates)	56.3	99.5 (1 in all isolates)
34	*hisB*	97.2–97.3 (29 in 1 O156:H8, 30 in 4 other O156:H8)	53.9	99.4 (2 in all isolates)
35	*hisC*	97.6 (26 in all isolates)	55.1	98.9 (4 in all isolates)
36	*hisD*	94.7 (69 in all isolates)	57.4	97.7 (10 in all isolates)
37	*hisG*	98.4–98.6 (13 in 4 O156:H8 isolates, 14 in 1 other O156:H8 isolate)	54.5	99.7 (1 in all isolates)
38	*hisL*	100	43.1	100
39	*yefM*	98.0 (5 in all isolates)	47.6	100
40	*yoeB*	99.6 (1 in all isolates)	44.1	100
41	*yeeZ*	99.5 (4 in all isolates)	51.6	99.3 (2 in all isolates)
42	*yeeY*	100	50.6	100
43	*yeeF*	100	52.7	100
44	*yeeE*	99.6 (4 in all isolates)	50.5	99.4 (2 in all isolates)
45	Hypothetical protein gene (1)	100	51.3	100
46	*sbcB*	99.9 (2 in all isolates)	50.7	99.8 (1 in all isolates)
47	*dacD*	98.4 (19 in all isolates)	49.3	99.0 (4 in all isolates)
48	*sbmC*	98.3 (8 in all isolates)	46.3	100
49	*yeeA*	97.9 (22 in all isolates)	50.3	99.1 (3 in all isolates)
50	Hypothetical protein gene (2)	99.7 (1 in all isolates)	47.7	100
51	*yoeF*	94.2 (7 in all isolates)	45.0	87.2 (5 in all isolates)
52	*cobU*	100	50.8	100
53	*cobS*	99.9 (1 in all isolates)	53.4	100
54	*cobT*	98.1 (21 in all isolates)	52.5	98.3 (6 in all isolates)
55	*erfK*	97.2 (26 in all isolates)	50.1	97.4 (9 in all isolates)
56	Transcriptional regulator, DeoR family gene	Missing in all O156:H8	None	None
57	Putative PTS IIA-like nitrogen-regulator protein PtsN gene	Missing in all O156:H8	None	None
58	PTS system, gelactitol-specific IIB component gene	Missing in all O156:H8	None	None
59	Sugar-phosphate isomerase, RpiB/LacA/LacB family gene	Missing in all O156:H8	None	None
60	Transcriptional regulator, DeoR family gene 2	Missing in all O156:H8	None	None
61	Putative PTS IIA-like nitrogen-regulatory protein PtsN gene 2	Missing in all O156:H8	None	None
62	PTS system, galactitol-specific IIC component gene	Missing in all O156:H8	None	None
63	Ribulose-phosphate3-epimerase gene	Missing in all O156:H8	None	None
64	Short-chain dehydrogenase/reductase SDR gene	Missing in all O156:H8	None	None
65	Sugar isomerase gene	Missing in all O156:H8	None	None
66	*nac*	98.7 (12 in all isolates)	48.7	99.7 (1 in all isolates)

The results presented herein imply that specific persistent STEC isolates can replace their O-AGC to change their phenotype. We postulate that STEC isolates that originally had the serotype O182:H25 changed their O-AGC to become STEC O156:H25 with sporadic O156:H8 isolates having served as potential DNA donors. An intraspecies switch in *E. coli* from O55:H7 to O157:H7 was previously described (Tarr et al., 2000; Wang et al., 2002). Another intraspecies gene exchange was demonstrated in *V. cholerae* (Blokesch and Schoolnik, 2007). Natural transformation following addition of genomic DNA from an O139 donor strain to a competent O1 strain growing as a biofilm on a chitin surface was sufficient for exchange of the O1-AGC against the entire O139-AGC in a single transformation event. Such O-antigen switches can play important roles in at least two steps of the infection process (Lerouge and Vanderleyden, 2002). They can affect colonization via altered adherence and the recombinant strains also have different antigenic properties, which confers a selective advantage as it allows the strains to bypass or overcome host defense responses (Bik et al., 1995). Compared to O1 strains, *V. cholerae* O139 variants, for example, are resistant to an O1 lytic phage (Blokesch and Schoolnik, 2007), colonize a mouse model with 2-fold higher efficiency (Waldor et al., 1994), are more invasive and damage the mucosal and submucosal layers more aggressively (Amin et al., 2009), and cause disease in persons with preexisting immunity to *V. cholerae* O1 (reviewed in Ramamurthy et al., 2003). In light of the epidemiological data presented herein and previously (Geue et al., 2002; Barth et al., 2016), it is tempting to assume that altered properties have also helped the STEC isolates studied to realize a more persistent lifestyle in the ruminant host.

The question arises how this O-AGC exchange occurred mechanistically. In *V. cholerae*, chitin-induced natural transformation can mediate the switch during a short period of time and with a high frequency (Blokesch and Schoolnik, 2007). *E. coli* has not been shown to be naturally competent (see Sinha and Redfield, 2012 and references cited therein), although several reports mention uptake of plasmid DNA under specific conditions (Baur et al., 1996; Tsen et al., 2002; Etchuuya et al., 2011; Guo et al., 2015). *E. coli* has homologs to competence genes from *Haemophilus influenzae* and they are expressed sufficiently to allow growth on DNA as sole carbon and energy source (Finkel and Kolter, 2001). If the *E. coli* transcription factor Sxy, whose homolog is indispensable for competence development in *H. influenzae*, and the lambda Red recombinase system are artificially expressed, *E. coli* can take up and incorporate foreign DNA into its genome (Sinha and Redfield, 2012). Natural conditions that induce the expression of *sxy* have not been identified so far. However, extraintestinal pathogenic *E. coli* isolates display higher recombination rates than commensal strains (Rodríguez-Beltrán et al., 2015). Higher recombination frequencies are positively associated with the presence of virulence factors (Rodríguez-Beltrán et al., 2015), suggesting that other *E. coli* pathovars might also display increased recombination activity. It will be interesting to study if this is the case for STEC isolates and if conditions promoting host colonization (La Ragione et al., 2009; Barnett Foster, 2013; Pacheco and Sperandio, 2015) can contribute to natural transformability of *E. coli*.

Another possibility for introducing foreign genetic material is generalized transduction by bacteriophages. In the EHEC strain EDL933, the *stx2AB* genes are located on prophage 933W, which is capable of transducing genetic markers in unmodified EHEC and *E. coli* K-12 strains (Marinus and Poteete, 2013). With ~27.4 kB, the length of the entire O-AGC encoding region from the O156:H25 isolate, which differs in sequence from the O182:H25 isolate, is well within the maximal amount of 61 kB that can be transferred by the phage. Despite all being negative for Stx phages (Barth et al., 2016), the O156:H8 isolates described in this study were originally isolated as *stx1* or *stx2* positive colonies (Geue et al., 2002). The loss of *stx* genes can already occur during the first subcultivation step and appears to be more frequent in non-O157 strains (Joris et al., 2011). It is therefore possible that either a lost *stx*-converting phage or other phages encoded in the O156:H8 genomes might have been involved in the generation of transducing phages containing the O-antigen region.

The O-antigen conversion proposed here for the two different serovars reinforces the importance of the O-antigen for host colonization and bacterial niche adaptation and adds another facet to the enormous genetic diversity and genomic plasticity of *E. coli* (Lukjancenko et al., 2010; Leimbach et al., 2013), again emphasizing the role of HGT in pathogen evolution. It also points to a probably overlooked aspect of *E. coli*/EHEC/STEC pathogenicity. Many different STEC serovars have been linked with human disease (Werber et al., 2008). The proposed HGT-mediated seroconversion suggests that additional genomic characterization of these isolates could reveal that they belong to only a limited number of sequence types each with its own set of specific virulence factors. Analysis of such “viro-STs” or “viro-clonal complexes” would aid the epidemiological analysis of disease outbreaks and pathogen evolution, help identify virulence factors either common to all or rather specific to only one or a few “viro-ST groups” aiding in their characterization and thereby improving our chances of finding and devising better strategies to combat STEC.

## Author contributions

LG, CM, LW, and SB designed the research; LG, IE, DP, CB, and SB performed the research; LG, SB, CB, and TS analyzed data; LG, CB, and CM wrote the paper.

## Funding

This work, including the efforts of SB, CM, and LG was funded by Deutsche Forschungsgemeinschaft (DFG) (GE2509/1-1).

### Conflict of interest statement

The authors declare that the research was conducted in the absence of any commercial or financial relationships that could be construed as a potential conflict of interest.
